# FOXA1 inhibits hepatocellular carcinoma progression by suppressing PIK3R1 expression in male patients

**DOI:** 10.1186/s13046-017-0646-6

**Published:** 2017-12-06

**Authors:** Shujiao He, Junyi Zhang, Wan Zhang, Fengsheng Chen, Rongcheng Luo

**Affiliations:** 1 Cancer Center, Southern Medical University, Guangzhou, Guangdong 510315 China; 2 Integrated Hospital of Traditional Chinese Medicine, Southern Medical University, Guangzhou, Guangdong 510315 China

**Keywords:** FOXA1, Hepatocellular carcinoma, PIK3R1/PI3Kp85, PI3K/Akt

## Abstract

**Background:**

Forkhead box A1 (FOXA1) expression is associated with various types of tumors; however, the function and underlying mechanism of FOXA1 in the development of hepatocellular carcinoma (HCC) remains obscure.

**Methods:**

Here, we investigated the role of FOXA1 in the development of HCC by applying gene function gain and loss analysis to HepG2 and Hep3B cell lines, and comparing outcomes with those of clinical HCC samples.

**Results:**

Phosphoinositide-3-kinase regulatory subunit 1 (PIK3R1), which encodes protein PI3Kp85 (p85), was identified as a FOXA1 target gene. Analyses of the mechanism and function revealed that FOXA1 suppresses hepatocellular carcinoma cell viability and motility by inhibiting PI3K/Akt signaling through direct inhibition of PIK3R1 transcription. Moreover, in clinical samples from male HCC patients, FOXA1 expression was much lower, whereas PI3Kp85 levels were much higher in tumor than in non-tumor tissues. Elevated PI3Kp85 is an unfavorable factor in HCC.

**Conclusions:**

As a tumor suppressor, FOXA1 targets PIK3R1 directly to inhibit PI3K/Akt signaling pathway, thus exerting a negative regulatory effect on proliferation, migration, and invasion of HCC in male patients.

**Electronic supplementary material:**

The online version of this article (10.1186/s13046-017-0646-6) contains supplementary material, which is available to authorized users.

## Background

Hepatocellular carcinoma (HCC) is one of the deadliest cancers worldwide [1, 2]. According to data published by the International Agency for Research on Cancer in 2012, over 78 million new cases of HCC and more than 70 million deaths due to HCC are recorded per year. As HCC is a highly heterogeneous disease, several genes and proteins are known to contribute to its tumorigenesis and progression [3].

Forkhead box A1 (FOXA1), also called HNF3A [4], is a member of the forkhead family of DNA-binding proteins, which are known for their role in regulating metabolism. FOXA proteins include three members, FOXA1, FOXA2, and FOXA3, each encoded by an individual gene [5]. Increasing evidence indicates that FOXA factors are involved in the development and progression of several tumors [6].

In the present study, we focused on FOXA1 as a transcriptional regulator of HCC. Function gain and loss analysis was performed to determine the role of FOXA1 in cancer cells derived from male HCC patients.

## Methods

### Cell culture

Human liver carcinoma cell lines HepG2 and Hep3B, both derived from male hepatocellular carcinoma patients according to ATCC, were purchased from the Cell Bank at the Chinese Academy of Sciences (Shanghai, China). According to the manufacturer’s instructions, the cells were cultured in Minimum Essential Medium (MEM; Gibco, Thermo Fisher Scientific, Waltham, MA, USA) supplemented with 10% fetal bovine serum (FBS; Gibco), penicillin (100 U/mL), and streptomycin (100 μg/mL) at 37 °C in a humidified atmosphere containing 5% CO_2_.

### Cell transfection

Cells were seeded in 6-well plates (1.5 × 10^5^ or 3 × 10^5^ cells/well) and maintained in complete medium for 12 h prior to transfection with siRNA (GenePharma, Shanghai, China) or plasmid DNA (Vigene Bioscience Inc., Shandong, China). siRNA sequences are available in Additional file 1: Table S1. siRNA and the FOXA1 plasmid were transfected into HepG2 and Hep3B cells at working concentrations of 100 nM using Lipofectamine 3000 (Invitrogen, Thermo Fisher Scientific, Waltham, MA, USA) following the manufacturer’s protocol. Scrambled-sequence siRNA and the pEnter plasmid were transfected as negative controls. Cells were harvested after 48–72 h and used for further experiments.

### Lentivirus infection

HepG2 and Hep3B cells were infected with FOXA1-overexpressing lentivirus (GeneChem, Shanghai, China), after which FOXA1 expression was confirmed by quantitative reverse transcription polymerase chain reaction (qPCR) and western blotting.

### Quantitative reverse transcription PCR

Trizol reagent (Takara Bio Inc., Shiga, Japan) was used to extract RNA from the cells and total RNA was then reverse transcribed according to the manufacturer’s protocol. PCR cycling conditions were 95 °C for 30 s to denature the cDNA template, followed by 40 cycles at 95 °C for 5 s and 60 °C for 20 s. The specificity of amplification products was confirmed by melting curve analysis. Data were analyzed using the 2^−ΔΔCt^ method. The primers used for qPCR are available in Additional file 1: Table S2. Independent experiments were performed in triplicate.

### Western blotting

Cells were lysed using RIPA lysis buffer (Beyotime, Shanghai, China) supplemented with phenylmethylsulfonyl fluoride and phosphatase inhibitors (Roche, Basel, Switzerland). After the protein concentration was determined with the BCA kit (Beyotime), cell lysates were subjected to SDS polyacrylamide gel electrophoresis and the protein bands were transferred to polyvinylidene fluoride membranes. The membranes were then probed with the following primary antibodies: anti-FOXA1 (ab170933; Abcam, Cambridge, UK), PI3Kp85 (ab86714; Abcam), anti-Akt (2920; Cell Signaling Technology, Danvers, MA, USA), anti-phospho-Akt (Ser473, 12,694; Cell Signaling Technology), anti-glyceraldehyde 3-phosphate dehydrogenase (RM2002; Beijing Ray Antibody Biotech, Beijing, China), and anti-Flag (F1804; Sigma, St. Louis, MO, USA). Bands were visualized using horseradish peroxidase-conjugated secondary antibodies and electrochemiluminescence detection kit. All western blot images were processed by ImageJ software (https://imagej.nih.gov/ij/).

### Cell proliferation assays

For the Cell Counting Kit-8 (CCK-8) assay, transfected HepG2 and Hep3B cells were seeded in 96-well plates at a density of 10^3^ cells/well. For transient transfection with siFOXA1 and siPIK3R1, cells were cultured for 1, 2, 3, 4, or 5 days. For lentivirus-mediated FOXA1 overexpression, cells were incubated for 1, 2, 3, 4, 5, 6, or 7 days. Subsequently, 100 μL of complete medium supplemented with 10 μL of CCK-8 solution (Dojindo, Kumamoto, Japan) was added to each well, the plates were incubated for 2 h, and the absorbance was measured at 450 nm.

EdU assays: Transfected HepG2 and Hep3B cells were seeded in 96-well plates at a density of 4 × 10^3^ cells/well. After incubation with 10 mM EdU (RiboBio, Guangzhou, China) for 2 h, cells were fixed and stained according to the manufacturer’s protocol. EdU-positive cells were counted under a fluorescence microscope in five random fields.

Colony formation assays: Lentivirus-infected HepG2 and Hep3B cells were seeded in 6-well plates at a density of 300 cells/well and cultured for 14 days. Subsequently, colonies were fixed with 100% methanol and stained with 1% crystal violet. Colonies composed of more than 50 cells in a well were counted under a microscope. All experiments were performed three times.

### Migration and invasion assay

For the transwell migration or invasion assay (8.0 μm, #3442; Corning, Corning, NY, USA), 1 × 10^5^ treated cells were seeded into the upper chamber in the presence of an uncoated or a Matrigel-precoated membrane (356,234; Corning) containing 200 μL of serum-free MEM. Complete medium (600 μL) containing 10% FBS was added to the bottom chamber. Following incubation for 24–30 h, the chambers were washed twice with phosphate-buffered saline, fixed with 100% methanol, and stained with 1% crystal violet at room temperature. Cells were counted under a microscope in five random fields.

### Chromatin immunoprecipitation (ChIP) assay

To examine whether FOXA1 bound to the promoter sequence of PIK3R1, a ChIP assay (17–371; Millipore, Merck, Darmstadt, Germany) was performed following the manufacturer’s protocol. Briefly, untreated Hep3B cells were fixed using 1% formaldehyde for 10 min to crosslink proteins to DNA, and then soluble chromatin was sheared into 200–1000 bp fragments using sonication. The fragmented chromatin samples were incubated with anti-FOXA1 antibody (ab23738; Abcam) to precipitate the putative binding sequences. Finally, PCR was used to detect enrichment of PIK3R1 promoter fragments on the putative FOXA1 binding sites. Primers used in ChIP are available in Additional file 1: Table S3.

### Dual luciferase reporter assay

Hep3B cells were plated at 2 × 10^5^ cells/well in 24-well tissue culture plates. PIK3R1-promoter-pGL3-basic plasmid (Kidan Bio Co. Ltd., Guangzhou, China), pRL-TK plasmid (Kidan Bio Co. Ltd), and FOXA1-pEnter plasmid (Vigene Bioscience Inc.) or pEnter (Vigene Bioscience Inc.) vector were co-transfected into Hep3B cells. After culturing for 48 h, luciferase activity was measured using the Dual-Luciferase Reporter Assay System (Promega Corporation, Madison, WI, USA).

### Immunohistochemistry assay

Immunohistochemistry assays were employed to detect expression of FOXA1 and PI3Kp85 proteins in paraffin-embedded human tissue microarrays (Shanghai Outdo Biotech, Shanghai, China), using a standard immunoperoxidase staining procedure and anti-FOXA1 (1:100, ab170933; Abcam) and anti-PI3Kp85 (1:50, ab86714; Abcam) antibodies. Stained tissue sections were examined separately by two pathologists. Protein expression was evaluated in terms of the proportion and intensity of stained cells. Thus, positive cells were scored based on their staining proportions as 1 (< 25%), 2 (26–50%), 3 (51–75%), and 4 (> 75%); and in terms of intensity as negative (0), weak (1), medium (2), or strong (3).

### Computational analysis of putative target genes regulated by FOXA1

Putative target genes controlled by FOXA1 were identified in silico analysis using the Cistrome Dataset Browser (http://cistrome.org/db/#/) [7] and ChIPBase v2.0 (http://rna.sysu.edu.cn/chipbase/) [8] databases on ChIP-seq data from HepG2 cells. Genes that scored higher than 2.5 points or encoded proteins were selected for further analysis using the Cistrome Dataset Browser and ChIPBase v2.0, respectively. Gene Ontology (GO) enrichment analysis was employed to cluster predicted genes from the datasets.

### Statistical analysis

SPSS 20.0 software (SPSS Inc. Chicago, IL, USA) was used for statistical analyses. Values represent the mean ± standard error of the mean of at least three independent experiments. Comparisons between two groups were performed using Student’s t-test. Multi-way classification analysis of variance was performed for the results of the CCK-8 assays [9]. Associations between FOXA1 and PI3Kp85 were analyzed using Spearman’s correlation coefficient. Survival analysis was performed using the Kaplan-Meier method. All statistical tests were two-sided, with statistical significance defined as *P < 0.05, **P < 0.01, and ***P < 0.001.

## Results

### FOXA1 suppresses viability and motility of liver carcinoma cells

**Fig. 1:**
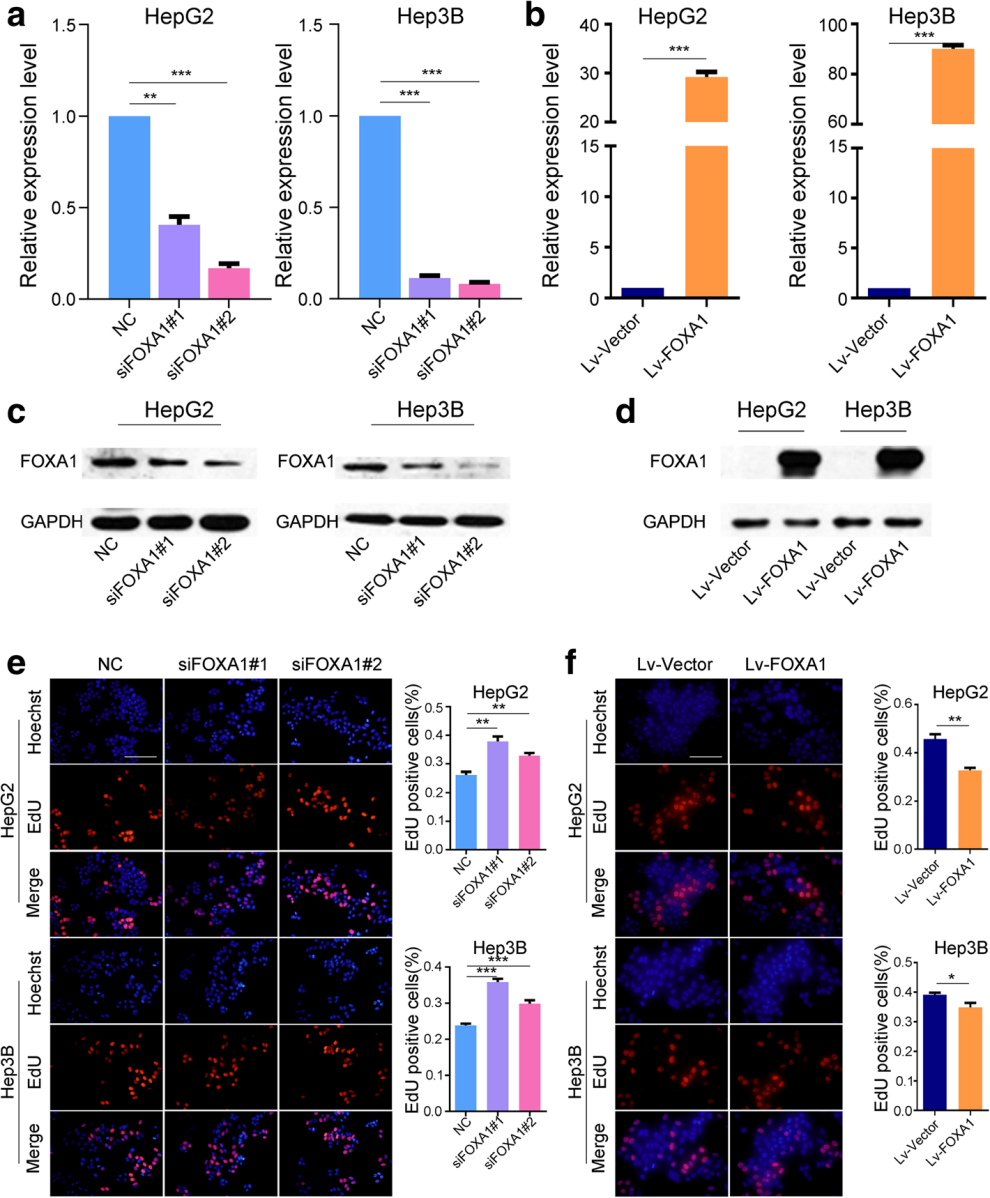
FOXA1 inhibits HCC cell proliferation. **a**-**d** Downregulation and upregulation of FOXA1 in HepG2 and Hep3B cells were confirmed using qPCR (**a**, **b**) and western blot (**c**, **d**). GAPDH was used as a loading control. EdU assay showed that FOXA1 downregulation increases cell proliferation (**e**), whereas upregulation of FOXA1 significantly decreases proliferation (**f**) (EdU magnification ×200)

**Fig. 2:**
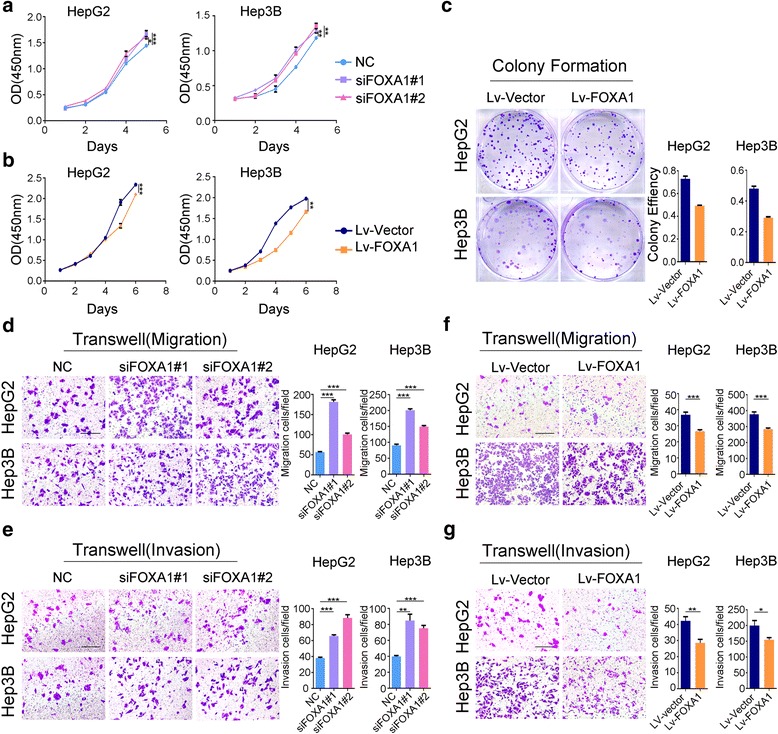
FOXA1 inhibits HCC cell viability and mobility. Decreased expression of FOXA1 promoted cell proliferation in CCK-8 analyses (**a**), whereas increased expression of FOXA1 reduced cell proliferation as shown by CCK-8 (**b**) and colony formation assays (**c**). Transwell migration (**d**, **e**) and invasion (**f**, **g**) suggest that inhibition of FOXA1 affects cell mobility (Transwell magnification ×200)

To investigate the role of FOXA1 in HCC development, we first investigated the effect of FOXA1 on cell viability and motility in HepG2 and Hep3B cells. HepG2 and Hep3B cells were transduced with siFOXA1 or FOXA1-overexpressing lentiviruses, respectively. To ensure whether the FOXA1 protein expression were consistent with the mRNA expression levels, FOXA1 downregulation and upregulation were confirmed at both mRNA and protein levels through qPCR (Fig. 1a–b) and western blot analysis (Fig. 1c–d) analysis, respectively. Subsequently, the effect of decreased and increased FOXA1 levels on cell proliferation was analyzed using CCK-8, colony formation, and EdU incorporation assays (Figs. 1e–f and 2a–c). Significantly more HepG2 and Hep3B cells were observed following transfection with siFOXA1#1 and siFOXA1#2 than with scramble siRNA. Conversely, FOXA1 overexpression markedly suppressed cell proliferation.

We then investigated the effect of FOXA1 expression on invasion and migration of HepG2 and Hep3B cells in vitro, using a transwell chamber coated with or without Matrigel. FOXA1 downregulation enhanced cell migration and invasion, whereas FOXA1 overexpression blocked HepG2 and Hep3B cell migration and invasion (Fig. 2d–g). Taken together, function gain and loss experiments in hepatocellular carcinoma cell lines revealed that downregulation of FOXA1 accelerated cell proliferation, migration, and invasion, whereas FOXA1 overexpression suppressed the viability and motility of the cells.

### FOXA1 directly targets PIK3R1

**Fig. 3:**
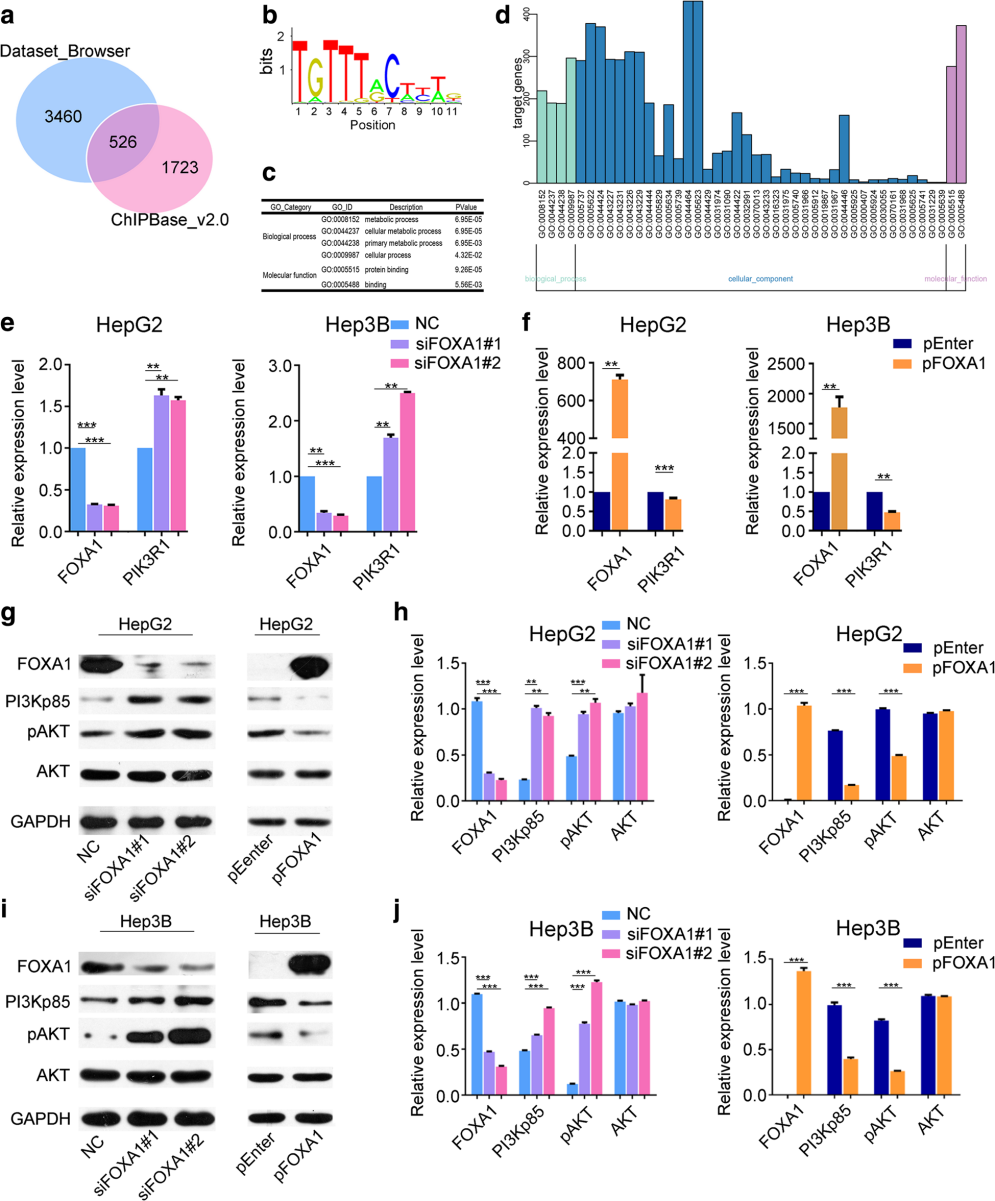
FOXA1 regulates the expression of PIK3R1. Based on the FOXA1 conserved motif (**b**), we explored FOXA1 target genes in Cistrome dataset_browser and ChIPBase_v2.0 datasets (**a**) combined with GO enrichment analysis (**c**, **d**), and we selected our target gene of interest, PIK3R1. qPCR revealed that downregulation of FOXA1 decreased the PIK3R1 mRNA level, whereas plasmid overexpression of FOXA1 increased PIK3R1 expression (**e**, **f**). In HepG2 (**g**, **h**) and Hep3B (**i**, **j**) cells, decreased FOXA1 expression promoted PI3Kp85 expression, thereby increasing PI3K/AKT activity, whereas increased FOXA1 expression suppressed PI3K/AKT activity through PI3Kp85 suppression. GAPDH was used as the loading control

To explore the mechanism underlying FOXA1 suppression of HCC cell proliferation, migration, and invasion, we searched for FOXA1 target genes in HepG2 cells in Cistrome Dataset Browser and ChIPBase v2.0 databases. In total, 3460 protein-coding genes scoring higher than 2.5 points and 1723 protein-coding genes with significant differences (*P* < 0.05) were obtained from the Cistrome Dataset Browser and ChIPBase v2.0 databases respectively. Thereafter, 526 FOXA1 target protein-coding genes were collected from the intersection of these 3460 and 1723 genes (Fig. 3a–b). GO enrichment analysis was performed using Cytoscape/BiNGO software to further categorize the 526 FOXA1 target genes. We noticed that PIK3R1 emerged in all of the biological process and molecular function subgroups of GO with significant differences (Fig. 3c–d). Until now, it was reasonable to predict that PIK3R1 was a direct target of FOXA1.

To evaluate the regulatory effect of FOXA1 on PIK3R1, changes in mRNA and protein levels of PIK3R1 were assessed following knockdown or overexpression of FOXA1. Per the qPCR results, PIK3R1 mRNA levels were elevated through FOXA1 downregulation (Fig. 3e), and reduced through FOXA1 overexpression (Fig. 3f) in HepG2 and Hep3B cells. However, western blot analyses also revealed that FOXA1 protein levels were negatively correlated with PI3Kp85 protein levels (Fig. 3g–j) in vitro. Furthermore, phosphorylation of Akt protein increased after silencing of FOXA1, but decreased after FOXA1 overexpression (Fig. 3g–j).

**Fig. 4:**
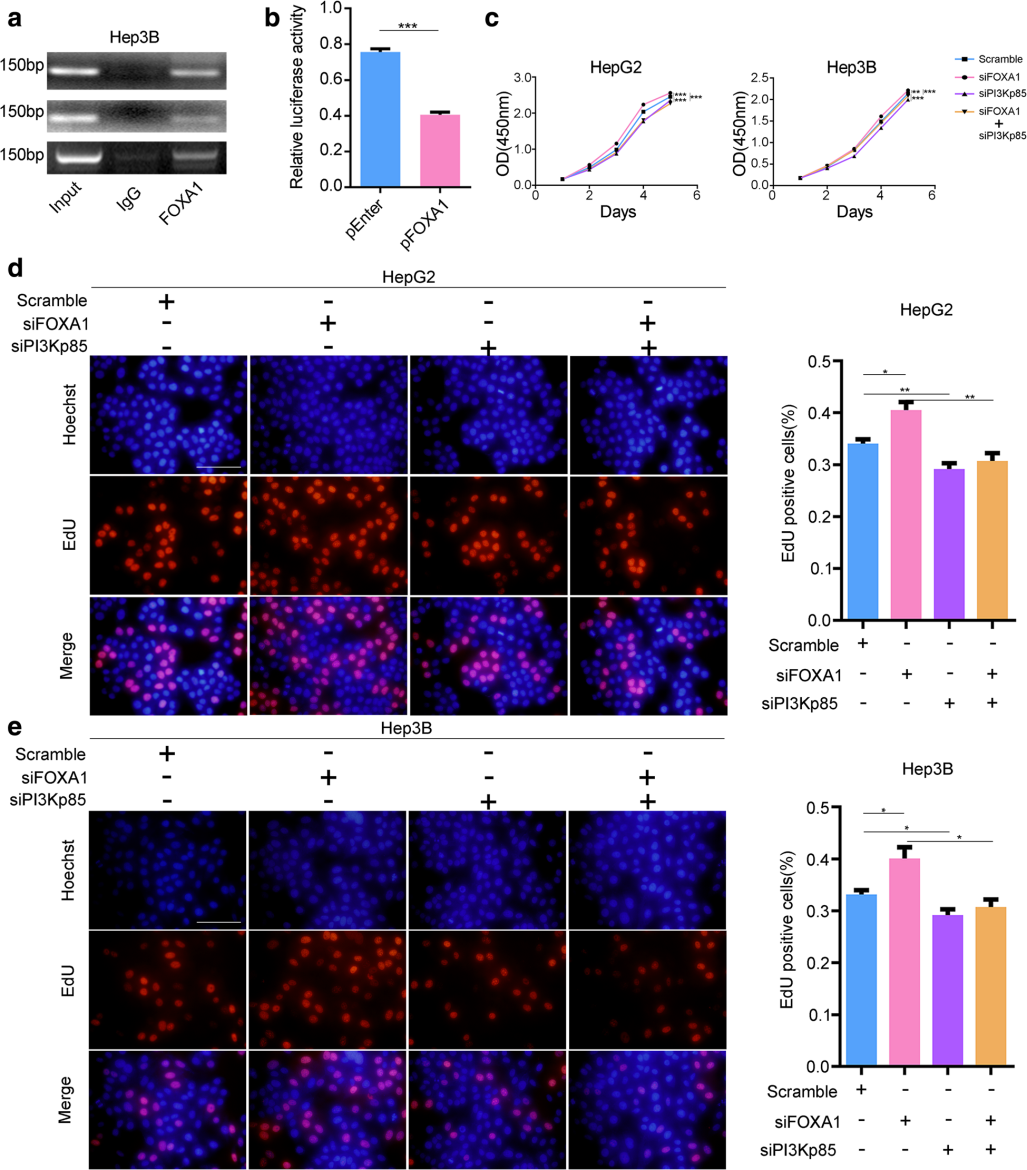
FOXA1 directly inhibits PIK3R1 transcription. FOXA1 directly binds to the PIK3R1 promoter sequence (**a**) and suppresses PIK3R1 transcription (**b**). Knockdown of PI3Kp85 reverses the increase in cell proliferation induced by downregulation of FOXA1 (**c**-**e**) (EdU magnification ×400)

To confirm whether FOXA1 protein directly regulated the transcription of PIK3R1, ChIP and dual luciferase reporter assays were performed using Hep3B cells. Based on the conserved motif of FOXA1 (Fig. 3b), the JASPAR database (http://jaspar.genereg.net/) was used to predict the binding sites of FOXA1 on PIK3R1 promoter sequence. Three sites in the promoter sequence of PIK3R1 were identified and combined with FOXA1 protein through ChIP-PCR (Fig. 4a); furthermore, dual luciferase reporter assays confirmed that FOXA1 directly inhibited PIK3R1 transcription by binding with the promoter sequence of PIK3R1 (Fig. 4b).

In summary, we proved that FOXA1 could directly repress PIK3R1 transcription, thus inhibiting signaling through the PI3K/Akt pathway in HepG2 and Hep3B cells.

### PIK3R1 knockdown restores cell viability and motility following FOXA1 downregulation

**Fig. 5:**
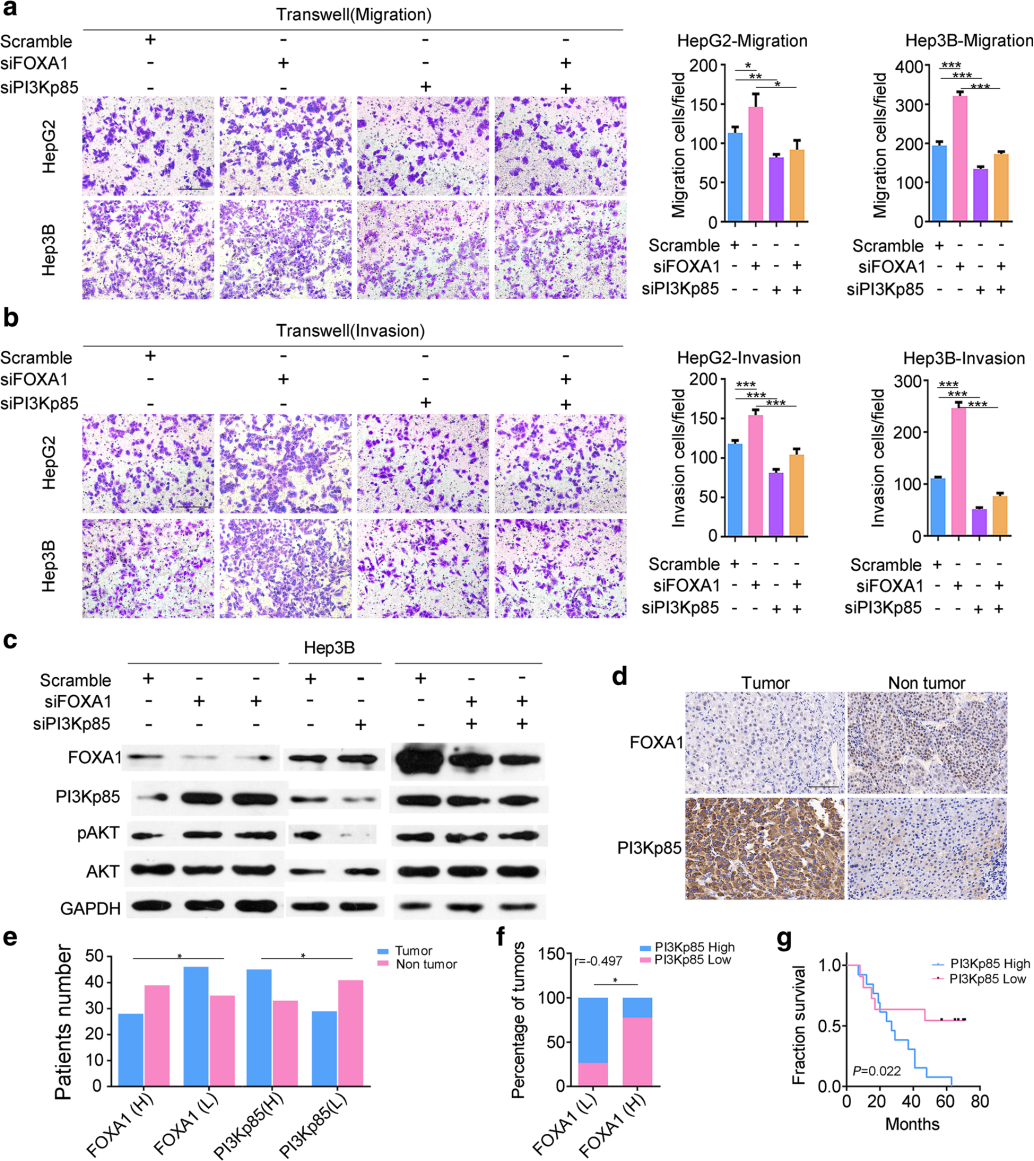
Inhibition of PI3Kp85 reduces cell mobility. (**a**, **b**) Transwell migration and invasion assays show that simultaneous knockdown of FOXA1 and PI3Kp85 decreased cell migration and invasion, whereas pAKT levels were decreased compared with knockdown of only FOXA1 (**c**); GAPDH was used as the loading control. In male HCC tissue, the FOXA1 level was much lower than that in matched non-tumor tissue, whereas PI3Kp85 was highly elevated in HCC tissue (**d**, **e**). In stage II male HCC patients, FOXA1 was negatively correlated with PI3Kp85 (**f**), and low expression of PI3Kp85 predicted a favorable outcome (**g**) (transwell and IHC magnification ×200)

HepG2 and Hep3B cells transiently co-transfected with siFOXA1 and siPI3KP85 exhibited lower cell proliferation, as revealed by CCK-8 and EdU incorporation assays (Fig. 4c–e). Furthermore, PIK3R1 downregulation significantly inhibited cell migration and invasion as revealed by the transwell assay (Fig. 5a–b). These results indicate that PI3Kp85 knockdown counteracted the increase in cancer cell viability and motility induced by FOXA1 downregulation. Additionally, the levels of phospho-Akt were found to be decreased in co-transfected cells (Fig. 5c).

### Pathoclinical features of FOXA1 and PI3Kp85 expression in patients with HCC

Finally, expression of FOXA1 and PI3Kp85 proteins was assessed using a commercial tissue microarray, with paired counterparts, of 90 patients with HCC. The clinical pathological factors and expression of FOXA1, PI3Kp85 in HCC are provided in Additional file 1: Table S4. In male patients, FOXA1 levels were significantly higher in precancerous tissue than in matched HCC tissue; conversely, PI3Kp85 levels were much lower in non-tumor tissue than in tumor tissue (Fig. 5d–e); however, this expression pattern was not observed in female patients. Notably, in male patients with stage II HCC, FOXA1 expression was negatively correlated with PI3Kp85 expression (Fig. 5f and Additional file 1: Table S5), and patients with low expression of PI3Kp85 presented longer survival times (Fig. 5g).

## Discussion

FOXA1 has been linked to various types of tumors [10, 11]. Intriguingly, it exhibits a dual role even in the same pathological condition [12], partly owing to FOXA1 acting both as a pioneer factor and as a transcription factor [4, 13]. In the latter case, FOXA1 acts as a critical regulator of metabolism, tissue function, and tumor development. In this study, we investigated the role and molecular mechanism of FOXA1 in HCC. Our findings revealed that FOXA1 protein directly regulates transcription of PIK3R1, which encodes PI3Kp85, and blocks HCC proliferation, migration, and invasion. Consistently, in HCC patients, FOXA1 expression was negatively correlated with PI3Kp85 expression in male subjects, and low expression of PI3Kp85 was a favorable factor in stage II male patients with HCC. These results suggest that FOXA1 functions as a potential HCC suppressor.

According to previous studies, FOXA1 is a polytropic gene, often associated with sex hormones [4]. In estrogen receptor-positive breast cancer, prostate cancer [14], acute myeloid leukemia, and thyroid carcinoma [6], FOXA1 exhibits a potential cancer-promoting effect; however, it causes tumor inhibition in estrogen receptor-negative breast cancer [15], advanced prostate cancer, and pancreatic cancer [10]. Further molecular mechanistic studies have revealed that FOXA1 promotes tumor progression by recruiting other transcription factors, while acting as a transcription factor for suppressing tumor development by directly regulating target gene expression [12, 16]. Until now, studies on the role of FOXA1 in carcinogenesis have focused mainly on breast and prostate cancers. Although FOXA1 was first detected in the liver, its role in HCC remains unclear. Recently, Li et al. [17] demonstrated that the sexual dimorphism of HCC was reversed in Foxa1/Foxa2-deficient mice. Mostly, though, it is believed that FOXA1 and FOXA2 do not interact with each other [18]. The exact mechanism by which FOXA1 regulates HCC progression remains poorly understood.

The PI3K/Akt signaling pathway is well known for mediating fundamental carcinogenic processes, including cancer cell survival, differentiation, proliferation, and motility [18]. The PI3K/Akt signaling pathway is constitutively activated in nearly all cancer types, probably through activation of upstream signaling molecules [19] or mutation of pathway components [20]. PI3K is composed of a regulatory subunit, PI3Kp85, and a catalytic subunit, PI3Kp110 [21], whose combination determines the biological activity of PI3K [22]. Recent studies have suggested that PI3Kp85 may act as an oncogene in several tumor types [23, 24], but the role of PI3Kp85 in HCC remains unclear.

In this study, function loss and gain experiments showed that FOXA1 knockdown induced proliferation, migration, and invasion of HepG2 and Hep3B cells, whereas FOXA1 overexpression decreased cell viability and motility. Moreover, the biological functions of FOXA1 identified in this study provide a mechanistic explanation for its role in carcinogenesis. We obtained the FOXA1 putative target gene, PIK3R1, by retrieving information from combined ChIP databases and GO enrichment analysis. Based on the FOXA1 conserved motif, we predicted its binding sites on the PIK3R1 promoter, designed eight pairs of primers to validate the possibility of a successful combination, and observed at least three binding sites. Simultaneously, the luciferase reporter assay indicated that FOXA1 functions as a transcriptional inhibitor of PIK3R1. The reduction in phospho-Akt activity by FOXA1 was mediated by PI3Kp85 and was involved in the PI3K/Akt signaling pathway, as determined by western blotting.

Finally, we confirmed a negative correlation between FOXA1 and PI3Kp85 in stage II male HCC patients, but not in female patients. Moreover, stage II male patients with HCC with low PI3Kp85 were predicted to have a long survival time. This might be linked to the origin of our clinical samples, which were collected from patients with HCC, who underwent surgery, and were in the early stage of the disease. Additionally, it should be noted that both HepG2 and Hep3B cell lines were constructed using samples from Caucasian male patients. These limitations emphasize the importance of investigating the regulatory effects of FOXA1 on PIK3R1 in female patients and in those diagnosed with HCC at different stages. For example, the Mahlavu cell line could be used to investigate how FOXA1 functions in female patients in vitro and more specimens from female patients should be collected to verify the role of FOXA1 in female patients.

## Conclusions

In conclusion, this study establishes an important role for FOXA1 in HCC, particularly in male patients. By negatively regulating PIK3R1 transcription, FOXA1 inhibits cell proliferation, migration, and invasion in male patients.

## Additional file

Additional file 1: Table S1. siRNA used in this study. **Table S2** Primers used in qPCR. **Table S3** Primers used in ChIP analysis. **Table S4** Correlation between clinical pathological factors and expression of FOXA1, PI3Kp85 in HCC **Table S5** Specimens exhibit low or high FOXA1 expression in relation to PI3Kp85 expression. (DOCX 25 kb)

## Abbreviations

ATCC: American type culture collection
ChIP: Chromatin immunoprecipitation
GO: Gene ontology
HCC: Hepatocellular carcinoma
PIK3R1: Phosphoinositide-3-kinase regulatory subunit 1
