# Supplementary material for: FOXA1 inhibits hepatocellular carcinoma progression by suppressing PIK3R1 expression in male patients
Source: J Exp Clin Cancer Res. 2017 Dec 6;36:175. doi: 10.1186/s13046-017-0646-6 (PMC5718070; doi:10.1186/s13046-017-0646-6)
Supplement: Additional file 1: Table S1. — siRNA used in this study. Table S2 Primers used in qPCR. Table S3 Primers used in ChIP analysis. Table S4 Correlation between clinical pathological factors and expression of FOXA1, PI3Kp85 in HCC Table S5 Specimens exhibit low or high FOXA1 expression in relation to PI3Kp85 expression. (DOCX 25 kb) [file 13046_2017_646_MOESM1_ESM.docx]

Additional file1

Table 1: siRNA used in this study

| siRNA | Sequence | |
| --- | --- | --- |
| siFOXA1#1 | Sense | GCACUGCAAUACUCGCCUUTT |
|  | Antisense | AAGGCGAGUAUUGCAGUGCTT |
| siFOXA1#1 | Sense | CCGGUCAGCAACAUGAACUTT |
|  | Antisense | AGUUCAUGUUGCUGACCGGTT |
| siPIK3R1 | Sense | GCUCGUGGAAGCCAUUGAATT |
|  | Antisense | UUCAAUGGCUUCCACGAGCTT |

Table 2: Primers used in qPCR

| Gene | Sequence | |
| --- | --- | --- |
| FOXA1 | F | GCTACTACGCAGACACGCAGGA |
|  | R | GGGTTGGCATAGGACATGTTGA |
| PIK3R1 | F | CAGCAACCTGGCAGAATTACGA |
|  | R | TGACAGGATTTGGTAAGTCCAGGAG |
| β-actin | F | TGGCACCCAGCACAATGAA |
|  | R | CTAAGTCATAGTCCGCCTAGAAGCA |

Table 3: Primers used in ChIP analysis

| PIK3R1-promoter | Sequence | |
| --- | --- | --- |
| Pair 1 | F | CTAGTGATATGGGGAGATATCATCAG |
|  | R | TTCCCAAATGCACCTCCTC |
| Pair 2 | F | GGTGTGAAGAAGGTACCTTGGT |
|  | R | AGCTCCACTTTAGGCTATTCAGAC |
| Pair 3 | F | ACGTGGTGCTGGCATTCCAT |
|  | R | CGGGGAAGCGCAGGTGAAT |

Table 4: Correlation between the clinical pathological factors and expression of FOXA1, PI3Kp85 in HCC

| Gene | Tissue | Expression  level | Gender | | Age | | Stage | |
| --- | --- | --- | --- | --- | --- | --- | --- | --- |
|  |  |  | female(N,%) | male(N,%) | ＞50(N,%) | ≤50(N,%) | Ⅰ(N,%) | Ⅱ(N,%) |
| FOXA1 | Tumor | H | 6（18.7） | 28（18.9） | 17（17.7） | 17（20.2） | 25（20.5） | 9(15.5) |
|  |  | L | 10（33.3） | 46（31.1） | 31（32.3） | 25（29.8） | 36（29.5） | 20(34.5) |
|  | Non-tumor | H | 3（9.3） | 39（26.3） | 23（23.9） | 19（22.6） | 29（23.8） | 13(22.4) |
|  |  | L | 13（40.6） | 35（23.6） | 25（26.0） | 23（27.3） | 32（26.2） | 16(27.6) |
| x value | | | 1.391 | 3.300 | 1.543 | 0.194 | 0.532 | 1.172 |
| *p* value | | | 0.217 | 0.049 | 0.150 | 0.413 | 0.292 | 0.209 |
| PI3Kp85 | Tumor | H | 11(34.3) | 45(30.4) | 28(29.2) | 28(33.3) | 40(32.8) | 16(27.6) |
|  |  | L | 5(15.6) | 29(19.6) | 20(20.8) | 14(16.7) | 21(17.2) | 13(22.4) |
|  | Non-tumor | H | 8(25) | 33(22.3) | 21(21.9) | 20(23.8) | 25(20.5) | 16(27.6) |
|  |  | L | 8(25) | 41(27.7) | 27(28.1) | 22(26.2) | 36(29.5) | 13(22.4) |
| x value | | | 1.166 | 3.903 | 2.043 | 3.111 | 7.409 | 0.000 |
| *p* value | | | 0.236 | 0.035 | 0.110 | 0.061 | 0.050 | 0.604 |

Table 5: Specimens exhibit low or high FOXA1 expression in relation to PI3Kp85 expression

|  |  | FOXA1(L) | | FOXA1(H) | |
| --- | --- | --- | --- | --- | --- |
| Gender | Stage | p85(L)  N | p85(H)  N | p85(L)  N | p85(H)  N |
| Female | Ⅰ | 3 | 2 | 0 | 6 |
|  | Ⅱ | 2 | 3 | 0 | 0 |
| Male | Ⅰ | 16 | 15 | 2 | 17 |
|  | Ⅱ | 4 | 11 | 7 | 2 |
